# Electrospun PVA/CS/HA/BA Nanofiber Scaffolds with Enhanced Mechanical Stability and Antifungal Activity for Bone Tissue Engineering

**DOI:** 10.3390/ma19020412

**Published:** 2026-01-20

**Authors:** Yagizer Yavuz, Ilyas Kartal, Sumeyye Cesur, Zehra Kanli, Elif Kaya, Gulgun Tinaz, Oguzhan Gunduz

**Affiliations:** 1 Department of Metallurgical and Materials Engineering, Institute of Pure and Applied Sciences, Marmara University, Istanbul 34722, Turkey; 2 Gedik Vocational School, Istanbul Gedik University, Istanbul 34913, Turkey; 3 Center for Nanotechnology & Biomaterials Application and Research (NBUAM), Marmara University, Istanbul 34854, Turkeyzehraknli@gmail.com (Z.K.);; 4 Department of Metallurgical and Materials Engineering, Faculty of Technology, Marmara University, Istanbul 34854, Turkey; ilyaskartal@marmara.edu.tr; 5 Department of Biochemistry, Faculty of Pharmacy, Health Sciences Institute, Marmara University, Istanbul 34854, Turkey; 6 Department of Basic Pharmaceutical Sciences, Faculty of Pharmacy, Marmara University, Istanbul 34854, Turkey

**Keywords:** *Candida albicans*, scaffold, cross-linking behavior, antifungal scaffolds

## Abstract

In this study, we created multifunctional bone tissue engineering scaffolds that combine prophylactic antifungal action with structural support. We produced PVA/CS/HA/BA nanofiber matrices via a specifically designed electrospinning technique to stop early cross-linking. Through SEM, our examination of fiber shape revealed diameters ranging from 178 ± 53 nm to 330 ± 69 nm. We discovered that this variation was closely correlated with the Boric Acid (BA) level. Our EDS and FTIR studies further showed that HA and BA were effectively mixed, with a specific focus on the production of borate-ester linkages inside the network. Mechanical examination revealed that 0.25 wt.% BA maximizes the tensile strength at 9.15 MPa, thereby closely matching HA-reinforced standards, while HA incorporation improved thermal stability. Moreover, in vitro hFOB experiments showed sustained cytocompatibility at 0.25 wt.% BA. While 0.5 wt.% BA showed strong antifungal action against *Candida albicans*, it sadly harmed cell viability. The 0.25 wt.% BA concentration ultimately offers a better balance between mechanical integrity and antibacterial action, therefore presenting a potential method for scaffold generation for bone regeneration in immunocompromised patients.

## 1. Introduction

Dealing with the medical challenges of non-union fractures and major bone loss calls for a unique synergy between biochemistry, cell therapy, and biomaterials [1]. Bone is often regarded only as a structural support system, but its biological relevance is far more extensive, as it is involved in essential homeostatic activities such as mineral storage and hematopoiesis [2]. In many cases of severe trauma or disease, the body’s own healing mechanisms reach a breaking point, where natural repair is no longer adequate. Tissue engineering comes in at this point, using bioactive signals and tailored scaffolds to help patients recover from surgery [3]. Recent technological developments have fueled a fast growth of this discipline’s regenerative limits.

Bone tissue is a highly dynamic system that consists of a complex of organic and inorganic components. Although collagen fibers are predominant within the organic phase, it has been observed that the inorganic phase is dominated by calcium phosphate components such as Hydroxyapatite (HA) [4]. Although bone defects are frequently caused by bacteria, fungal osteomyelitis, especially when brought on by *Candida* species, offers a serious and sometimes disregarded biomedical concern [5]. Particularly at risk are immunocompromised people and those on long-term antibiotic treatment. Fungal colonization of bone scaffolds can promote biofilm development, which results in implant failure and postponed healing. Poor bone penetration and major side effects are among the frequent problems of current systemic antifungal treatments. Including a prophylactic chemical such as Boric Acid (BA) straight into the scaffold therefore offers a very important local barrier against pathogens. Preventing fungal colonization at the source guarantees a cleaner regenerative environment; concurrently, BA’s ability to improve the mechanical strength of the scaffold is exploited [6,7,8]. On a cellular or microscopic scale, bone tissue is continuously renewed, and this is regulated by osteoblasts (cells that form bones), osteoclasts (cells that degrade bones), and osteocytes (mature bone cells that are embedded in bone matrices) [9]. Cell interaction and cooperation play a very significant role in bone regeneration. In tissue engineering, it is of the utmost importance that this cell synergy is achieved for successful outcomes. Bone tissue engineering materials can be broadly categorized into natural polymers, synthetic polymers, and ceramics [9]. While collagen, chitosan (CS), and gelatin are notable materials used as natural polymers, Polyvinyl Alcohol (PVA), Poly(Lactic-co-glycolic acid) (PLGA), and Polycaprolactone (PCL) are notable materials used as synthetic polymers, and HA is a notable ceramic material that possesses an osteoinductive ability due to its compatibility with the bone matrix. Recent innovations in bone tissue engineering have focused on developing sophisticated and functional scaffold systems by integrating biotechnology and materials science. Among various platforms, electrospun nanomaterials have emerged as highly effective controlled-release systems that facilitate bone healing by enhancing cell adhesion, proliferation, and subsequent tissue formation [9,10,11,12].

Thus, bone tissue engineering has emerged as a multidisciplinary area that holds great promise for bone regeneration through an integrated and harmonious blend of biomaterials, cell therapies, and growth factors [3]. Future research is expected to make significant contributions to the development of more targeted and effective therapeutic approaches.

HA is a calcium phosphate mineral, and it is widely applied in bioengineering [13]. Since it is considered optimal as a matrix for bone healing, HA is used to interact with bone tissue and support osteointegration because of its cytocompatibility and biodegradability [14]. Even though HA has low strength, its combinations with other polymers have been considered as potential ways of increasing its strength and applications [15].

Natural polysaccharide, CS, comes from the exoskeletons of shellfish [16]. While CS is naturally biodegradable and cytocompatible, its combination with HA enhances osseointegration by producing a synergistic composite system that supports bone development [17]. Moreover, CS’s inherent porosity and cell-adhesive qualities give the best microenvironment for cell attachment and growth. Because CS efficiently supports the biological needs of regenerating tissue, these features make it a very valuable biomaterial for bone tissue engineering applications.

PVA, a different well-known polymer in bioengineering, is sought after for its natural cytocompatibility, water solubility, and low toxicity. Incorporated with CS into a composite system, PVA greatly improves the water retention capability and mechanical strength of a substance. Moreover, electrospun nanofibers that are stabilized by hydrogen bonding and Van der Waals forces help to make hydrogels that are physically cross-linked. With their great structural integrity and cytocompatibility, these materials are very appropriate for many different uses in regenerative medicine and tissue engineering [18,19,20,21].

BA is regarded as a flexible agent with wide use in the antifungal and antibacterial fields. Particularly against *Candida* species and other microbial organisms, its great efficacy against a broad spectrum of pathogens has been shown in a significant amount of research [22,23,24,25]. BA has proven to be quite successful as an antifungal agent in both laboratory and biomaterial settings [25,26]. Electrospinning is widely used for producing nanofibers, and this is achieved through the spinning of a polymer solution or melt into a thin strand using a high voltage.

This technology involves extruding the polymer solution through a tip (needle or nozzle). Through electrostatic forces, the polymer solution is attracted, and a thin strand of liquid is created [27,28,29]. The strand is extended until it meets the collector surface, where it dries, resulting in nanofiber mats. Several factors influence the electrospinning process and the resulting nanofibers, including polymer concentration, applied voltage, flow rate, and collector distance [30,31,32]. The incorporation of biopolymers such as CS and bioceramics like HA into synthetic PVA matrices is highly valuable for improving cytocompatibility. However, while the addition of CS typically leads to a decrease in mechanical strength compared to pure PVA, the integration of HA serves to reinforce the nanofibrous structure [33,34,35].

Thus, combining HA, CS, PVA, and BA through electrospinning holds great potential for bone healing and repairing. Moreover, these composite materials have improved compatibility and strength, and they can be used effectively for biomedical applications [3]. Further studies have verified that electrospun nanofibers have great potential for achieving a successful drug-controlled release system for biomedical applications, and especially, the development of polymeric nanofiber mats concerning its mechanics and cytocompatibility is quite promising for potential biomedical applications [32].

The primary contribution of this research is the development of a multifunctional PVA/CS/HA/BA scaffold that addresses the functional need for antifungal protection in bone tissue engineering. While PVA/CS/HA systems are known for their osteoconductive properties, this work differentiates itself by utilizing BA as a dual-functional agent. BA acts as a molecular cross-linker that maintains the high mechanical stability provided by the HA (bioceramic) phase, while simultaneously providing potent antifungal activity against *Candida albicans*. Furthermore, we introduce a tailored solution processing strategy, achieving an optimization of nearly 150 h, to overcome the challenge of premature gelation (pre-cross-linking) in PVA-BA mixtures. This approach ensures the production of uniform nanofibers and offers a promising platform for developing cytocompatible scaffolds that are capable of preventing opportunistic fungal infections during bone regeneration.

The central scientific hypothesis of this study is that BA functions as a ‘Molecular Bridge’ that synergistically reinforces the interface between the organic PVA/CS matrix and the inorganic HA phase. As supported by previous studies on boron-mediated cross-linking behavior, the formation of dynamic borate-ester bonds between the hydroxyl groups of PVA/CS and the phosphate groups on the HA surface effectively bridges the organic–inorganic interface, enhancing the overall structural stability. This synergy allows the scaffold to maintain its mechanical integrity (9.15 MPa tensile strength) while simultaneously providing a targeted antifungal shield against *Candida* species.

## 2. Materials and Methods

### 2.1. Materials

PVA (Mw: 89–98 kDa, 99% hydrolyzed) was purchased from Sigma-Aldrich; CH (high molecular weight) was produced by Sigma-Aldrich; HA (Mw: 502.31 g/mol); phosphate-buffered saline (PBS, pH = 7.4), glutaraldehyde solution (GA, 50 wt.% Mw = 100.12 g/mol), acetic acid (glacial, %100 anhydrous for analysis), and phosphate-buffered saline (PBS, pH 7.4) were all purchased from Sigma-Aldrich (St. Louis, MO, USA). BA (Mw: 61.83 g/mol) was obtained from Merck Chemicals (Darmstadt, Germany). Triton X-100 (PEG t-octylphenyl ether (*n* = 9–10) (TX-100) (molecular weight: 647 g mol^−1^) was purchased from Biobasic (Markham, ON, Canada).

### 2.2. Preparation of Electrospinning Solution

In the solution preparation process, 10% (*w*/*v*) PVA and purified water were first mixed with a magnetic stirrer at a stirring speed of 320 rpm and 95 °C. Following the preparation of the PVA solution, a separate CS solution was prepared by dissolving 1% (*w*/*v*) CS in a 1% (*v*/*v*) aqueous acetic acid solution. This mixture was stirred at 150 rpm for 45 min at 60 °C using a magnetic stirrer to ensure complete dissolution. Subsequently, the PVA and CS solutions were blended at a 9:1 (*v*/*v*) ratio and stirred for an additional 30 min at 60 °C (150 rpm). Finally, 50 mg of HA was incorporated into 10 mL of the resulting PVA/CS blend to achieve a final HA concentration of 0.5% (*w*/*v*).

BA, an antifungal agent, was then added. Here, during the mixing of PVA and BA for solution, it was observed that they cross-linked with the previously mentioned sequence during the preparation of the solution, and as a result, a gel-like structure emerged. Optimization parameters of BA addition were investigated to reduce the interactions between PVA and BA; the ideal parameters were determined through an optimization process of approximately 150 h. Firstly, 0.5% (*w*/*v*) HA was added to purified water and prepared at 70 °C and 300 rpm. After that, BA was adjusted so that its concentration in the solution did not exceed 1.5% (*w*/*v*) [36]. The solution was stirred with a magnetic stirrer at 70 °C and 300 rpm. After the HA-BA solution was prepared, for the addition of 10% (*w*/*v*) PVA, the speed of the magnetic stirrer was increased to create a vortex in the solution at a speed between 500 and 750 rpm, and thus PVA was added gradually. Then, the cross-linking reaction was prevented by reducing the rotational speed to 320 rpm for the first 5 min at 95 °C for 1 h and then at high rpm for the first 5 min. Based on the preliminary experimental findings, a cross-shaped magnetic stir bar was used in this study, as it resulted in less formation of cross-linkages relative to the others.

CS was then prepared as previously mentioned and added at a ratio of 9:1. With this process, the solution temperature was reduced to 60 °C. Then, it was mixed with a magnetic stirrer at a rotational speed of 150 rpm for 30 min and made suitable for electrospinning. In the spin processes, it was observed that blockages occurred at the needle tip, and as a result, problems occurred in the spin.

**Figure 1:**
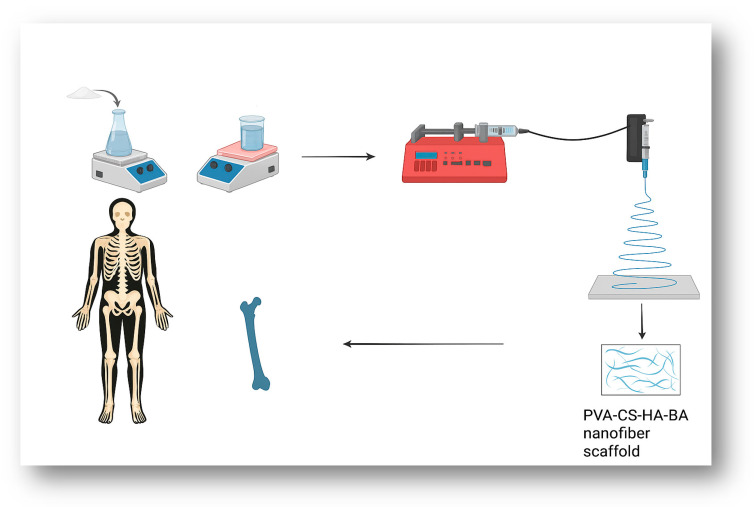
Systematic overview of preparation of PVA/CS/HA/BA nanofiber scaffolds.

Then, following related studies, Triton X-100 (TX-100) was added as a surfactant to reduce the surface tension and improve the electrospinnability of the polymer blend [37,38]. As a result, 5 (*w*/*v*) % TX-100 was added after the solution was prepared and stirred at 150 rpm and 60 °C for about 45 min. The addition of TX-100 successfully decreased the solution viscosity, thereby facilitating a more stable and continuous electrospinning process (Figure 1 and Table 1). 

### 2.3. Preparation of Nanofibers by Electrospinning and Chemical Cross-Linking

Nanofibrous scaffolds were fabricated using a single-needle electrospinning apparatus (Inovenso, Istanbul, Turkey) equipped with a high-voltage DC power supply (0–30 kV) and a programmable syringe pump for precise control of the polymer solution flow rate.

The optimized PVA/CS/HA/BA solutions were loaded into 10 mL plastic syringes fitted with a 19-gauge stainless-steel needle. The electrospinning process was carried out under ambient laboratory conditions (25 ± 2 °C) without additional humidity regulation.

To achieve uniform and bead-free fiber formation, process parameters such as applied voltage and flow rate were selected according to the solution composition. The applied voltage ranged between 25 and 27 kV, while the solution flow rate varied from 1.0 to 1.6 mL·h^−1^. The needle-to-collector distance was maintained at 12 cm, and the spinning duration was fixed to about 3 h, ensuring the continuous deposition of homogeneous nanofiber mats.

A cellulose-based release paper was employed as the collector substrate instead of aluminum foil to facilitate the easy removal of the nanofiber mats without causing structural deformation. Following electrospinning, the obtained nanofibers were air-dried at room temperature to remove residual solvent and moisture before subsequent characterization. The electrospinning parameters were determined through extensive preliminary experiments to identify the optimal processing window that ensures Taylor cone stability and the production of bead-free nanofibers for each specific composition.

The suitable electrospinning parameters and composition details of each sample are summarized in Table 2.

In this study, nanofibers produced by the electrospinning method were subjected to chemical cross-linking with glutaraldehyde (GA) vapor prior to testing to ensure the morphological stability of the fibers and stabilize the polymer chains within the structure. For this process, 5 mL of GA and 5 mL of pure water were mixed in a glass Petri dish, and the prepared mixture was placed in a desiccator. The samples were kept in this environment at 40 °C for 2 h. To ensure the complete removal of residual GA and prevent potential cytotoxicity, the cross-linked scaffolds were aerated in a fume hood for 24 h. Subsequently, the samples were extensively washed three times with PBS (pH 7.4) for 15 min each to eliminate any unreacted GA molecules. Following the purification steps, the scaffolds were dried under vacuum.

After the cross-linking process was completed, the samples obtained were subjected to tensile testing, differential scanning calorimetry (DSC), Fourier Transform Infrared Spectroscopy (FT-IR), and Scanning Electron Microscopy (SEM) analyses. Through these analyses, the morphological and chemical–mechanical properties of the nanofibers were determined.

### 2.4. Scanning Electron Microscopy

SEM is often used to examine nanofibers, because it creates high-resolution pictures that let you carefully assess their morphology, surface shape, texture, and size distribution [39]. Because SEM has a lot of magnification and a wide field of view, it can be used to measure the diameter, size uniformity, porosity, and beading of fibers very accurately. These natural structural qualities are major elements that have a direct bearing on nanofibers’ performance in sensing, filtration, and tissue engineering uses [39,40].

SEM (EVO LS 10, Zeiss, Jena, Germany) was used to examine the surface morphologies of the produced nanofibers. Each scaffold was sputter-coated with a thin layer of gold and palladium (Au-Pd) for two minutes using a sputter coater (Quorum SC7620, Quarum Technologies, Laughton, UK) in order to guarantee electrical conductivity and reduce charging effects. Energy-dispersive X-ray spectroscopy (EDS) connected with the SEM system at an accelerating voltage of 10 kV was used to examine the elemental composition and the distribution of Ca, P, and B inside the PVA/CS/HA/BA matrix. To guarantee statistical dependability, 100 randomly chosen fibers from the SEM images were measured for each sample to provide mean fiber diameters.

### 2.5. Fourier Transform Infrared Spectroscopy (FT-IR)

FT-IR is one of the basic tools employed to investigate the chemical structure and molecular arrangement of nanofibers. The method identifies chemical functional groups within the nanofiber structure through the analysis of molecular vibrational motion of chemical bonds to facilitate the confirmation of polymer type and interaction between substances in the composite material [41,42]. The chemical properties were determined with the assistance of the FT/IR-4700 (Jasco, Tokyo, Japan) analytical tool. The tests were conducted at 23 °C room temperature. The scanning speed of 32 units with a resolution of 4 cm^−1^ within a wavelength range of 4000–400 cm^−1^ was employed.

### 2.6. Differential Scanning Calorimetry (DSC)

DSC is a proven thermal analysis technique used to identify nanofibers’ crystalline behavior and thermal changes. Along with the crystallinity index of polymer-based nanofibers, this method offers vital information on the glass transition temperature (Tg), melting temperature (Tm), crystallization temperature (Tc), and thermal degradation temperature (Td). Information on these aspects is indispensable, as they directly affect the mechanical strength and long-term usefulness of the scaffold [43,44]. Thermal investigations in this study were carried out using a Shimadzu DSC-60 (Kyoto, Japan) under regulated conditions. From 25 °C to 400 °C, each sample was heated at a steady rate of 20 °C per minute. About 2250 data points per sample resulted from this procedure, which allowed for a careful analysis of the phase transitions and thermal stability of the nanofibers.

### 2.7. Mechanical Characterization

Nanofibers have emerged as an important class of materials and are distinguished by their critical mechanical properties across a wide range of applications, including tissue engineering, filtration, and composite reinforcement. Particularly in scaffold structures, mechanical strength similar to that of bone tissue is expected; therefore, the mechanical properties must be comparable to those of bone [45,46,47]. In this context, to determine the mechanical properties of nanofibers, the thicknesses of the samples were measured (Mitutoyo America Corporation, Aurora, IL, USA), and then samples measuring 10 × 50 mm were taken and subjected to tensile testing using a tensile testing machine (Shimadzu Corporation, EZ-LX, Kyoto, Japan).

### 2.8. Cell Viability Evaluation of Nanofiber Scaffolds (Indirect MTT Assay)

The cytocompatibility properties of the fabricated nanofiber scaffolds were evaluated using human osteoblast cells (hFOB, CRL-3602™, ATCC, Manassas, VA, USA). Prior to the experiments, all nanofiber structures were sterilized under ultraviolet (UV) light for one hour on both sides. For the cell viability assessment, the MTT Cell Proliferation and Cytotoxicity Assay Kit (E-CK-A341) (Elabscience Biotechnology Co., Ltd., Wuhan, China) was used. As part of the indirect MTT method, nanofiber scaffold samples were prepared according to their respective groups and incubated in cell-free culture medium, allowing for the release of soluble components from the scaffolds into the medium. The resulting conditioned media were subsequently used in cell culture experiments.

hFOB cells were seeded into 96-well plates at a density of 5 × 10^3^ cells/well using DMEM-F12 medium supplemented with 10% fetal bovine serum (FBS) and 10,000 units/mL penicillin-streptomycin (conditioned medium). Each experiment was repeated in triplicate. The cells were cultured in a humidified incubator at 37 °C with 5% CO_2_. MTT assays were performed on days 1, 4, and 7 following cell seeding. On the specified days, fresh medium (100 µL) was added to each well, followed by the addition of 50 µL MTT stock solution. The plates were then incubated at 37 °C for 2.5 h. After incubation, the medium was removed, and 150 µL of DMSO was added to each well to dissolve the resulting formazan crystals. Cell viability was measured spectrophotometrically at 570 nm using a BioTek plate reader (Santa Clara, CA, USA).

### 2.9. Antifungal Activity Evaluation

The antifungal activity of the PVA-CS, PVA-CS-HA, PVA-CS-HA-BA-0.25, and PVA-CS-HA-BA-0.5 scaffold was tested by broth dilution method. *Candida albicans* Atcc 10,231 reference strain was used in the experiment. Before the experiment, *C. albicans* was cultured on SDA (Sabouraud Dextrose Agar) medium and left to incubation for 48 h. After incubation, the final concentration of fresh culture was adjusted to (0.5–2.5) × 10^5^ CFU/mL in RPMI 1640 medium, and then 48-well plates were added with scaffolds. The plates were incubated at 37 °C in a shaking incubator for 48 h. After incubation, absorbance was measured at OD625. The inhibition rate was calculated by comparison with the control *C. albicans* with no sample added (CLSI).

BI% = (ODC − ODS)/ODC × 100

Here, BI% is the inhibition of growth, ODC refers to the control *C. albicans* (absorbance value of the group without sample), and ODS is the absorbance value of the group of the sample.

### 2.10. Statistical Analysis

Data were presented as mean ± standard deviation (SD). All experiments were performed in triplicate (*n* = 3) to ensure statistical reliability. Statistical significance was determined using one-way analysis of variance (ANOVA), followed by Tukey’s post hoc test for multiple comparisons. All statistical analyses and graphical representations were performed using GraphPad Prism [10.3.1] software. A *p*-value of less than 0.05 was considered statistically significant. ** In the figures, significance levels were indicated as follows: * *p* < 0.05, ** *p* < 0.01, *** *p* < 0.001, and **** *p* < 0.0001. Differences with *p* > 0.05 were considered non-significant (ns).

## 3. Results and Discussion

### 3.1. Structural Morphology of Electrospun Scaffold System

The morphological characteristics of scaffolds are among the key factors affecting the performance of biomaterials used in tissue engineering. In this context, the surface morphology of PVA/CS/HA/BA nanofibers prepared by electrospinning was examined in detail by SEM to investigate the distribution of fiber diameter, pore morphology, and effects of additive materials (HA and BA) on nanofiber structure. Furthermore, energy-dispersive X-ray spectroscopy (EDS) was used to validate the incorporation and uniform distribution of HA and BA in the scaffolds. The results obtained confirmed the elemental composition of the nanofiber matrix.

The average diameter of pure PVA/CS nanofibers is 178 ± 53 nm, with a predominant diameter of 150–200 nm. This nanoscale size is remarkably close to the ECM of bone. Its applicability to tissue engineering can thus be verified [12].

The addition of HA did not cause a significant change in fiber diameters (178 ± 36 nm) but induced a slight roughness on the surface. This coarse morphology and high surface area have been deemed to be advantageous to osteoblast cells with respect to binding and proliferation. This is attributed to a homogeneous distribution of HA particles in the polymer matrix. EDS specifies the detection of Ca and P ions in the sample to confirm the above-mentioned distribution [12]. The incorporation of BA in the nanofibers’ processing results in considerable differences in their morphology and elemental composition. The incorporation of 0.25 wt.% BA caused the diameter to increase to 192 ± 49 nm and the size distribution to range between 100 and 400 nm. This is attributed to the enhancement of the viscosity of the solution with increased BA concentrations. Therefore, it is hypothesized that lower electrical conductivity causes a slowing in jet deposition speed. As a result, the fibers that form are a little thicker and have a bigger range of diameters [38,48,49]. Thicker fiber formation is well known to follow from greater viscosities.

**Figure 2:**
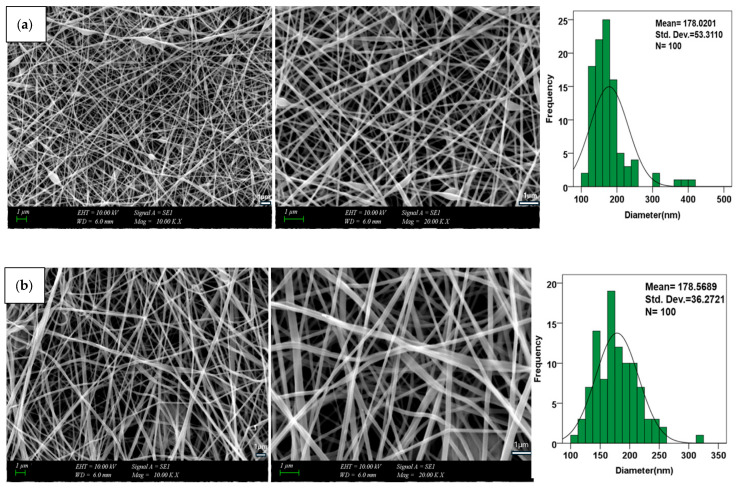

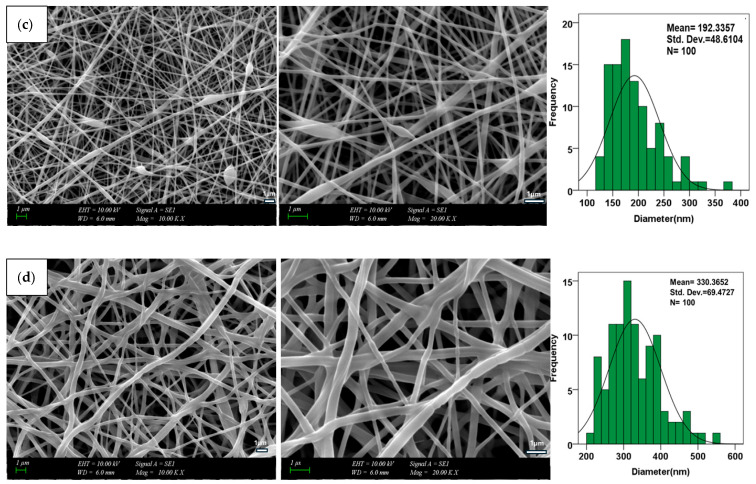
SEM micrographs showing the surface morphology and fiber diameter distribution of (**a**) PVA/CS, (**b**) PVA/CS/HA, (**c**) PVA/CS/HA/0.25BA and (**d**) PVA/CS/HA/0.5BA nanofiber scaffolds.

To stabilize the electrospinning process, the tip-to-collector separation was kept at 12 cm. As recorded in our preliminary findings in Table 2, this separation guaranteed Taylor cone stability and allowed enough flight time for full solvent evaporation, thus avoiding the fused fiber morphologies that are usually observed in the literature [32]. This setup clearly enables the jet thinning required to produce the consistent nanoscale dimensions and bead-free shape seen in Figure 2. Moreover, the solvent system is the primary factor shaping morphology outside of electrostatic characteristics. Particularly, the 1% (*v*/*v*) acetic acid solution encourages the protonation of chitosan’s amino groups, hence markedly raising the polymer jet’s net surface charge density. Under the electric field, this increased conductivity results in stronger uniaxial stretching, thereby producing the smaller and more uniform nanofibers seen in the SEM analysis [50].

When the BA concentration was increased to 0.5 wt.%, the average fiber diameter reached 330 ± 69 nm, and it was determined that the fibers were thicker, and their surfaces were more irregular. This trend can be explained by the hardening of the network structure due to the formation of dense borate-ester bonds between PVA chains at high BA concentrations and the decrease in the jet thinning ability during the electrospinning process [51] (Figure 2).

**Figure 3:**
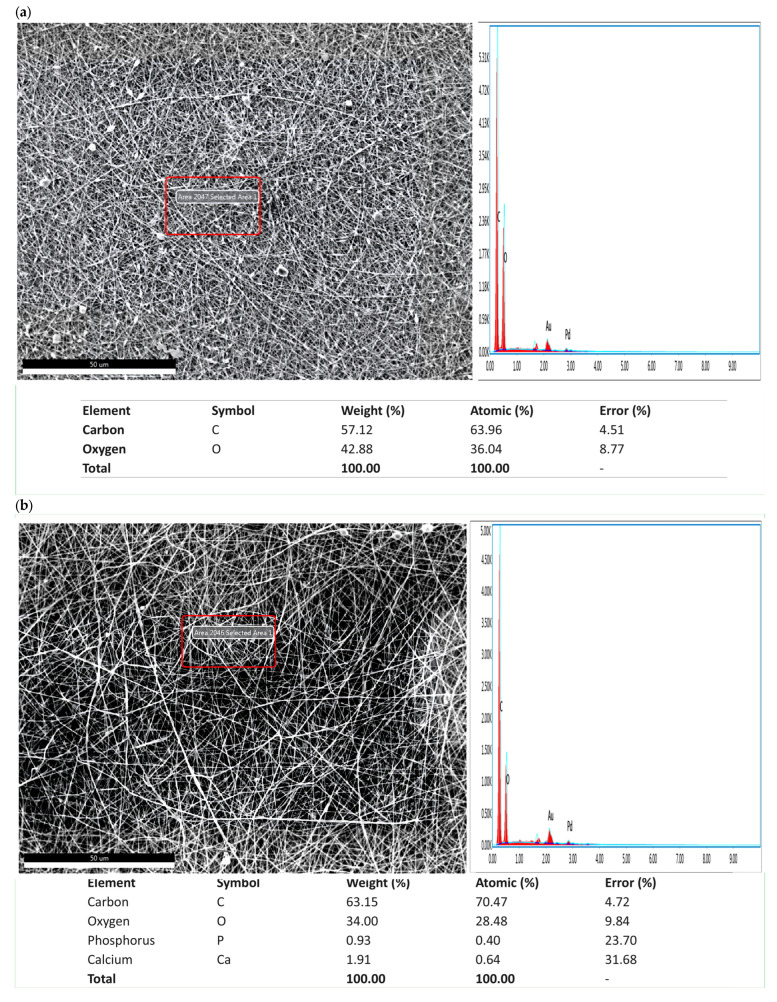

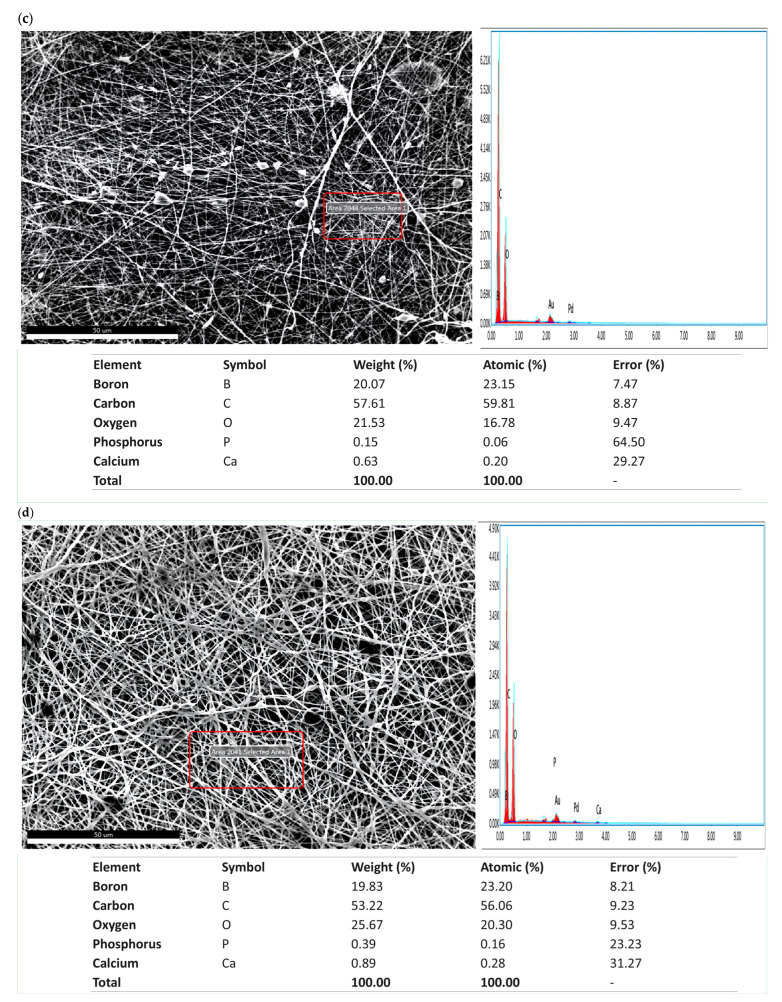
EDS analysis demonstrating the elemental distribution of electrospun PVA/CS/HA/BA nanofibers, confirming the incorporation of HA (Ca, P) and BA (B) within the composite matrix for samples: (**a**) PVA/CS, (**b**) PVA/CS/HA, (**c**) PVA/CS/HA/0.25BA, and (**d**) PVA/CS/HA/0.5BA.

These morphological findings were also supported by EDS analyses. The analyses revealed that the elemental composition of the fibers included Carbon (C) and Oxygen (O) elements, as well as boron (B) atoms, which are indicators of successful cross-linking. Thus, it has been confirmed that BA is chemically integrated into the polymer matrix and that the increase in diameter and morphological changes observed in SEM are the result of this chemical interaction [49] (Figure 3).

### 3.2. Spectroscopic Characterization and Molecular Interaction Analysis

**Figure 4:**
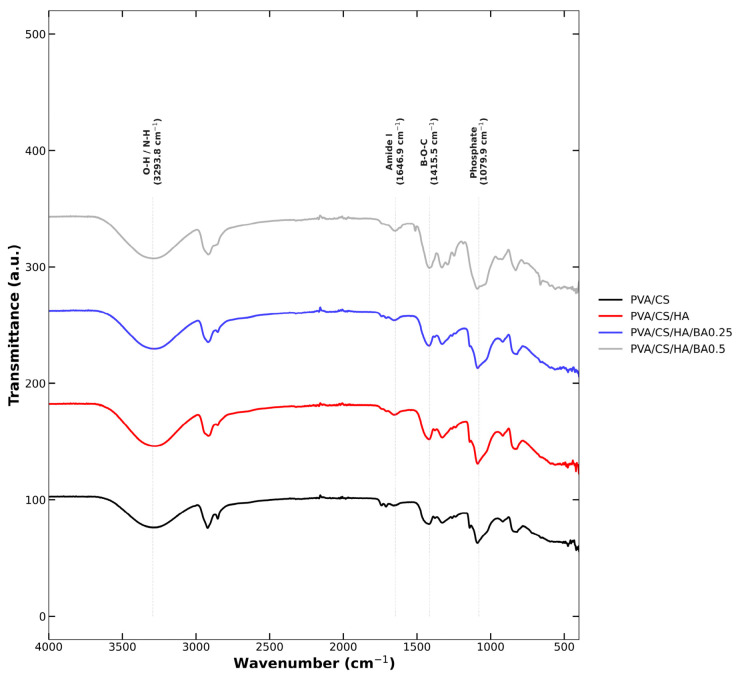
FTIR spectra of nanofibers. The spectra are shifted vertically (*Y*-axis offset) for clarity of visual comparison.

The FTIR results validated the incorporation of each material used to form the composites. For the PVA/CS composite, peaks related to O–H and N–H bonding (∼3300 cm^−1^), C–H bonding (∼2869–2989 cm^−1^) [52,53], and CS’s amide bonds (∼1592–1650 cm^−1^) [54,55] were identified. For the PVA/CS/HA composite, peaks corresponding to PO_4_^3−^ bonds (1030–1134 and 560–600 cm^−1^) were evident, validating the HA component [56]. For BA incorporation, peaks corresponding to B–O bonds at 1365–1367 cm^−1^ were identified. This was particularly weak at a low amount (0.25%) and predominant at a high molar amount (0.5%). The widening of the OH peak validates BA’s effects on the enhancement of H bonding. Moreover, it validates the rise in intermolecular forces among the PVA molecules within the pure PVA. These results confirm that the HA and BA were successfully integrated into the PVA/CS matrix. Previous studies related to similar PVA/CS/HA materials noted similar results for molar concentrations through similar PVA/CS-HA intermolecular interactions [57,58,59] (Figure 4).

The crystalline integrity of the incorporated HA was further evaluated via the resolution of phosphate vibrational modes in the FTIR spectra. The appearance of the PO_4_^3−^ doublet at 560 and 600 cm^−1^, combined with the high intensity of the peak at 1030 cm^−1^, is a strong indicator of the retention of the crystalline HA phase [11]. In amorphous or degraded phases, these peaks typically merge into broad, featureless bands. Although XRD analysis is a primary method for crystallinity determination, the high resolution of these specific phosphate vibrational modes provides definitive spectroscopic evidence of phase retention during the electrospinning process [56]. This spectral evidence, triangulated with the stoichiometric Ca/P atomic ratio (≈1.67) observed in EDS mapping (Section 3.1)—where the 0.25 wt.%BA group exhibited a calcium-rich surface (3.3) that facilitates cell attachment without intracellular toxicity, while the ratio approached the stoichiometric ideal (1.67) as the BA concentration increased (reaching 1.75 for 0.5 wt.% BA)—confirms that HA particles are crystalline but also homogeneously dispersed and structurally stable within the PVA/CS/BA matrix [11]. This spectral evidence, coupled with the uniform elemental distribution observed in EDS mapping, confirms that HA particles are homogeneously dispersed and structurally stable within the PVA/CS/BA matrix. As detailed in Table 3, the comparative intensity analysis of the FTIR spectra reveals critical insights into the molecular interactions within the scaffold. The characteristic peaks for borate-ester bonds (1365–1367 cm^−1^) displayed a marked increase in intensity as the BA concentration was raised from 0.25 to 0.5 wt.%, providing quantitative evidence of enhanced molecular cross-linking. Additionally, the significant broadening observed in the hydroxyl region (∼3300 cm^−1^) further confirms the synergistic development of a dense hydrogen-bonding network between the BA molecules and the PVA/CS matrix, which is consistent with the improved structural stability of the nanofibers (Table 3).

### 3.3. Thermal Stability and Transition Properties of Electrospun Scaffolds

From the DSC analyses, there is evidence of the role of additives on the thermal phenomena of PVA/CS-based nanofiber matrices. The peaks at approximately 63.8 °C in the pure PVA/CS sample can be attributed to the glass transition temperature (Tg) of the sample. Additionally, the endothermic peaks at 219–231 °C can be attributed to the partial melting of PVA and the beginning of chain dissociation. This is evidence that the semi-crystalline nature of PVA dominates thermal stability [68,69,70]. The CS additive prevents the disruption of the glass transition of the material and influences the thermal properties of the PVA. It is indicated in the literature that the 63.8 °C peak attributed to the glass transition temperature is conserved in the PVA/CS material [50,71,72]. The CS influences the role of the hydrogen bonds in the PVA matrix. As indicated in the literature, the CS’s amide groups determine the polymer’s interaction with heat during the DSC analysis. Presumably, it supports polymers’ thermal stability [73].

**Figure 5:**
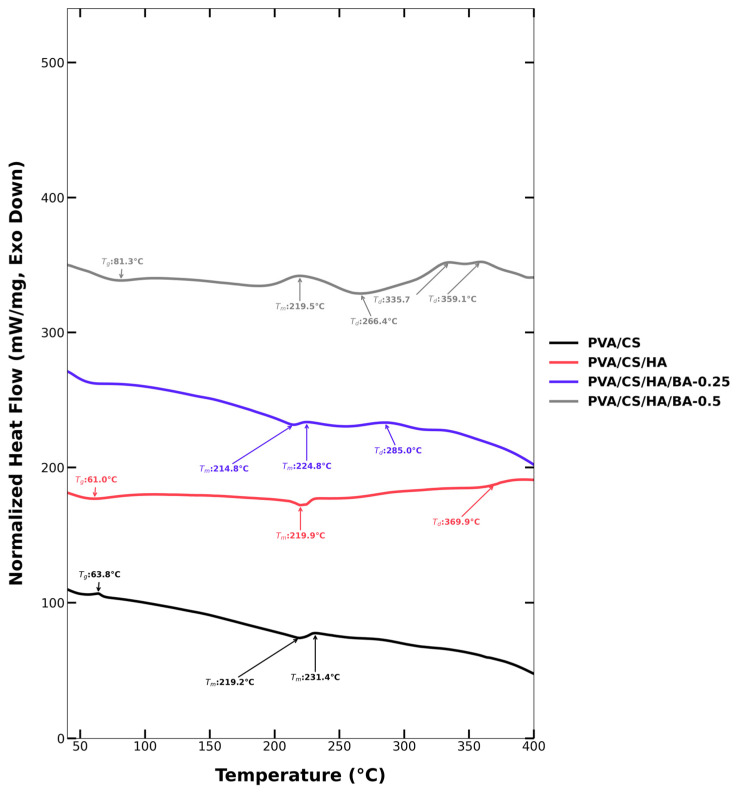
DSC thermograms of PVA/CS, PVA/CS/HA, PVA/CS/HA/0.25BA, and PVA/CS/HA/0.5BA nanofibrous scaffolds showing the thermal transitions and the effect of BA cross-linking on the glass transition and melting behavior.

With the addition of composite HA, the low-temperature peak related to Tg remained unchanged at approximately 61 °C. At the same time, the unique PVA melting peak at 219 °C remained. But there is another peak related to decomposition at 369.9 °C. This also indicates that decompositions at slightly higher temperatures have been achieved because of the partially stabilizing effects of HA on the polymer chain [74,75,76]. The effect of BA proved to be more critical. At a lower BA (0.25%) composition level, there were peaks around 214–285 °C. It is indicated that BA is involved in binding with polymer molecules to facilitate the enhancement of their hydrogen bonding. But there were peaks related to decompositions at the same time. This indicates that BA decomposed at slightly lower temperatures. The addition of BA at a high composition level (0.5%) resulted in a complex thermogram, with peaks ranging between 187 and 359 °C. At the same time, there is another peak related to Tg at 81 °C. This indicates that BA had a strong interaction with the polymer/HA matrix, because it has a cross-linking-agent-like effect, thus increasing thermal stability with a multistage degradation process [48,77]. Overall, the results obtained from the DSC experiments suggest that HA makes the composite more stable at high temperatures. At the same time, the BA additive causes a modification to the glass transition and decomposition processes. The thermal transition related to the results obtained from the experiments above, especially at high composition levels of BA, suggest that there is a strong interaction between the additive and the polymer matrix to physically and chemically modify it. This indicates that the nanofiber scaffolds have potential applications in providing stability and control over degradation for their potential uses in the field of biological applications(Figure 5).

The thermal behavior of the scaffolds is closely dictated by the segmental mobility of the polymer chains and the resulting degree of crystallinity. The introduction of BA facilitates the formation of a three-dimensional network through borate-ester linkages. These chemical junctions serve as ‘anchoring points’ that significantly restrict the long-range chain dynamics and decrease the free volume within the PVA/CS matrix. This molecular restriction is reflected in the observed shifts in thermal transition temperatures (Tg and Tm).

Furthermore, a measurable decrease in the melting enthalpy (*∆H*_m_) was observed in BA-containing groups, suggesting a reduction in the overall degree of crystallinity. This phenomenon occurs because the chemical cross-links act as structural defects that interfere with the orderly folding and close packing of PVA chains into crystalline lamellae. Consequently, while the cross-linked network enhances the overall thermal stability of the composite, the polymer matrix adopts a more disordered, amorphous-like state, which correlates with the improved structural integrity observed in the mechanical assessments.

### 3.4. Mechanical Properties of Nanofibers

The tensile strength value for the PVA/CS scaffold was calculated to be 3.16 ± 0.745 MPa, and the value of elongation at break was calculated to be 1.37 ± 0.557% [78,79]. The tensile strength value for the PVA/CS/HA nanofibers was calculated to be 9.24 ± 0.68 MPa, representing a significant enhancement compared to the pure polymer (****, *p* < 0.0001). In addition, the value of elongation at break was calculated to be 10.3 ± 2.01% (*, *p* < 0.05 vs. PVA/CS) [12]. In this case, HA was added to enhance the tensile strength by making them rigid. In PVA/CS/HA/0.25 BA-added nanofibers, the value of tensile strength was calculated to be 9.15 ± 0.58 MPa, and the value of elongation at break was calculated to be 8.95 ± 0.88%. In PVA/CS/HA/0.5 BA-modified nanofibers, the value of tensile strength was calculated to be 9.24 ± 0.51 MPa, as anticipated due to an increment in the BA content [51]. Statistical analysis via Tukey’s multiple comparisons test confirmed that there was no significant difference in tensile strength between the PVA/CS/HA group and the BA-added groups (*p* > 0.05), indicating that the incorporation of BA effectively preserved the mechanical reinforcement provided by the mineral phase.

**Figure 6:**
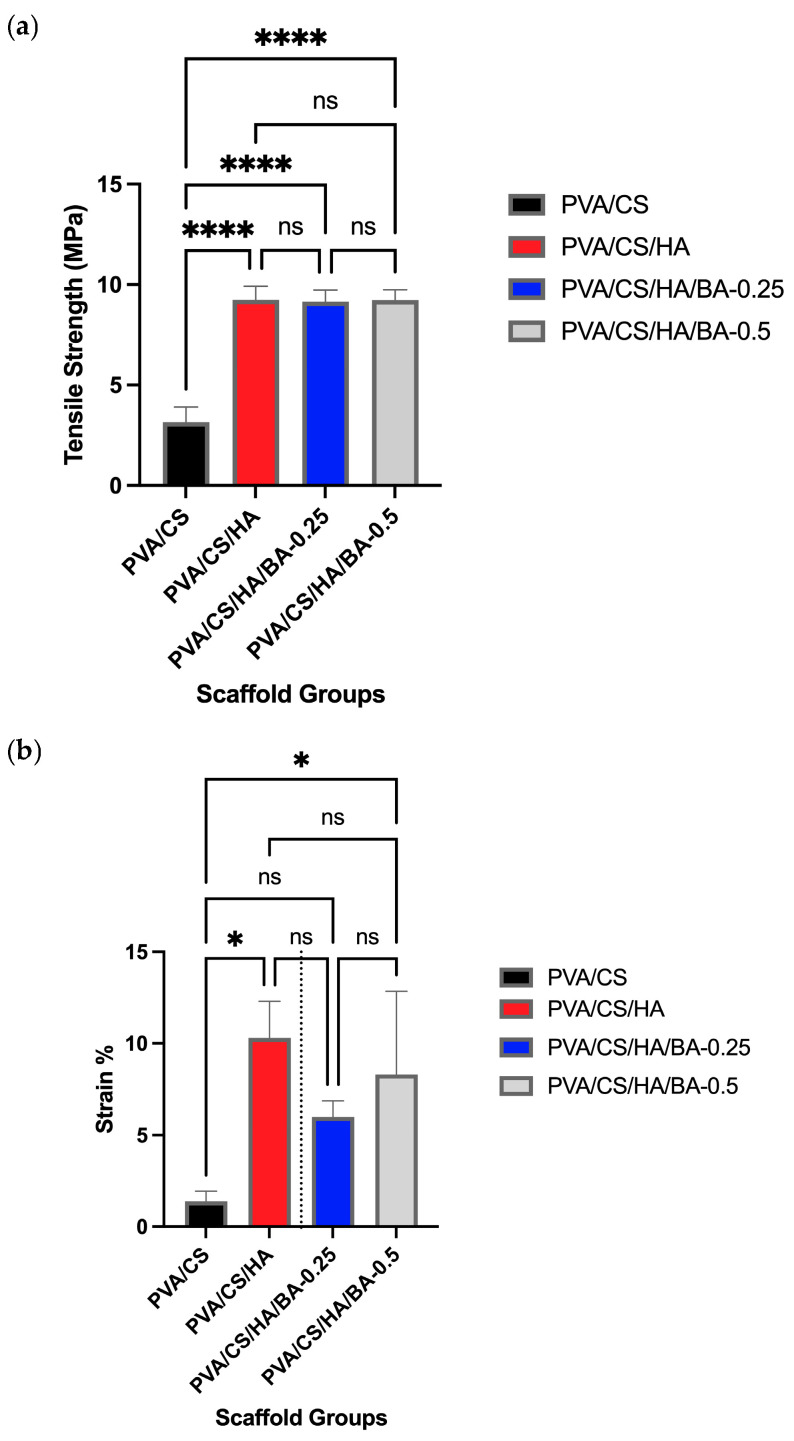
Mechanical properties of electrospun nanofiber scaffolds: (**a**) tensile strength (MPa) and (**b**) strain at break (%) of PVA/CS, PVA/CS/HA, PVA/CS/HA/0.25BA, and PVA/CS/HA/0.5BA samples. Data are presented as mean ± SD (*n* = 3). Statistical significance was determined using one-way ANOVA followed by Tukey’s post hoc test. Asterisks indicate significant differences: * *p* < 0.05 and **** *p* < 0.0001 compared to the PVA/CS group. ns indicates no significant difference (*p* > 0.05).

Also, the value of elongation at break was calculated as 8.31 ± 4.55%. There was a partial decrease in the value of elongation at break due to the BA additive, indicating that a partial decrease occurred within the structure due to the BA being present as an additive [51,80] (Figure 6). However, this decrease in elongation was statistically insignificant compared to the HA group (*p* > 0.05), confirming that the overall structural stability remained intact. In this case, for nanofibers with 0.25 BA, a partial decrease occurred in tensile strength value compared to that of PVA/CS/HA nanofibers, and this is due to lightly cross-linked and uncross-linked portions present. Statistical results confirmed that this numerical change was not significant (*p* > 0.05).

Together with the FT-IR analysis, these observations infer that borate ions have yielded more additional cross-links due to chemical complexation of hydroxyl groups, and this is due to an augmented feature of improved mechanics of structures [51,66].

The mechanical assessments were conducted in dry conditions as a benchmark for comparative analysis. However, the scaffolds were stabilized via GA vapor cross-linking to ensure structural integrity in physiological environments. The functional stability of the nanofibers was qualitatively confirmed during the 7-day cell culture studies, where the scaffolds remained intact in the aqueous medium, demonstrating their suitability for bone tissue applications despite the initial dry-state benchmarking.

### 3.5. Evaluation of the Cytocompatibility of Nanofiber Scaffolds

**Figure 7:**
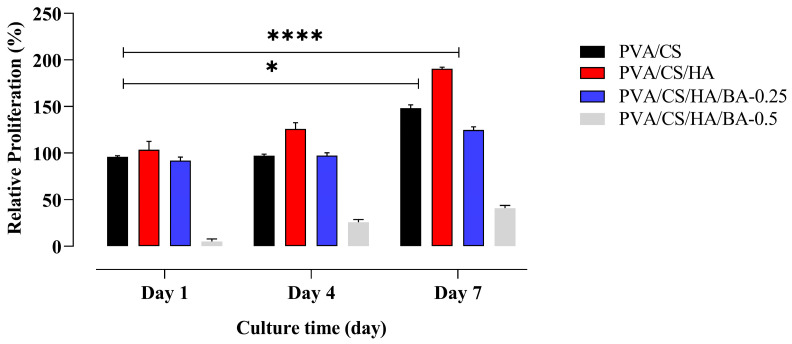
Cell viability of hFOB cells was assessed using the MTT assay. The viability of hFOB cells incubated with nanofibers was evaluated on days 1, 4, and 7. All experiments were performed in triplicate, and the data are presented as mean ± SD (*n* = 3). Asterisks indicate significant differences: * *p* < 0.05 and **** *p* < 0.0001 compared to the control group.

The MTT results show that although the cell viability in the PVA/CS group improved over time, this improvement was only somewhat modest. Particularly on days 4 and 7 (* *p* < 0.0447; **** *p* < 0.0001), a notable rise in cell viability was seen in the PVA/CS/HA group where HA was included, therefore underlining the positive impact of HA on cell proliferation and cytocompatability. On the other hand, the PVA/CS/HA/BA-0.5 group which had a greater concentration of BA (0.5) showed a clear reduction in cell viability at all time points, with strong cytotoxic effects becoming more noticeable as the incubation period went on. This suggests that a higher BA concentration compromises cytocompatibility and negatively affects cell survival. According to our initial dose–response studies, in which BA concentrations were methodically tested across a range from 0.75 to 1.5 wt.%, this outcome is consistent. This consecutive study showed that all concentrations inside this range were clearly and significantly cytotoxic, therefore confirming 0.5 wt.% as the unambiguous maximum biological limit for this scaffold system. On the other hand, cell viability remained high in the PVA/CS/HA/BA-0.25 group at all time intervals, demonstrating that BA at this concentration is not cytotoxic and maintains cytocompatibility. Even though the rise in cell viability was less than that of the HA-containing group, this small amount of BA offers a cytocompatible contribution that works well in the ‘therapeutic window,’ where antifungal effectiveness is balanced with the safety of the host cells. Finally, these findings support the general cytocompatibility of the produced composite scaffolds, therefore suggesting their possible use as safe and encouraging bases for tissue engineering and cell development applications (Figure 7).

To ensure that the observed biological responses were specifically due to BA concentration, all control and experimental scaffolds (PVA/CS, PVA/CS/HA, and PVA/CS/HA/BA) underwent an identical GA vapor cross-linking procedure. The high viability observed in BA-free control groups serves as an internal validation that residual GA was successfully eliminated and did not interfere with the cytocompatibility results.

### 3.6. Evaluation of Antifungal Activity of PVA/CS/HA/BA Scaffolds

**Figure 8:**
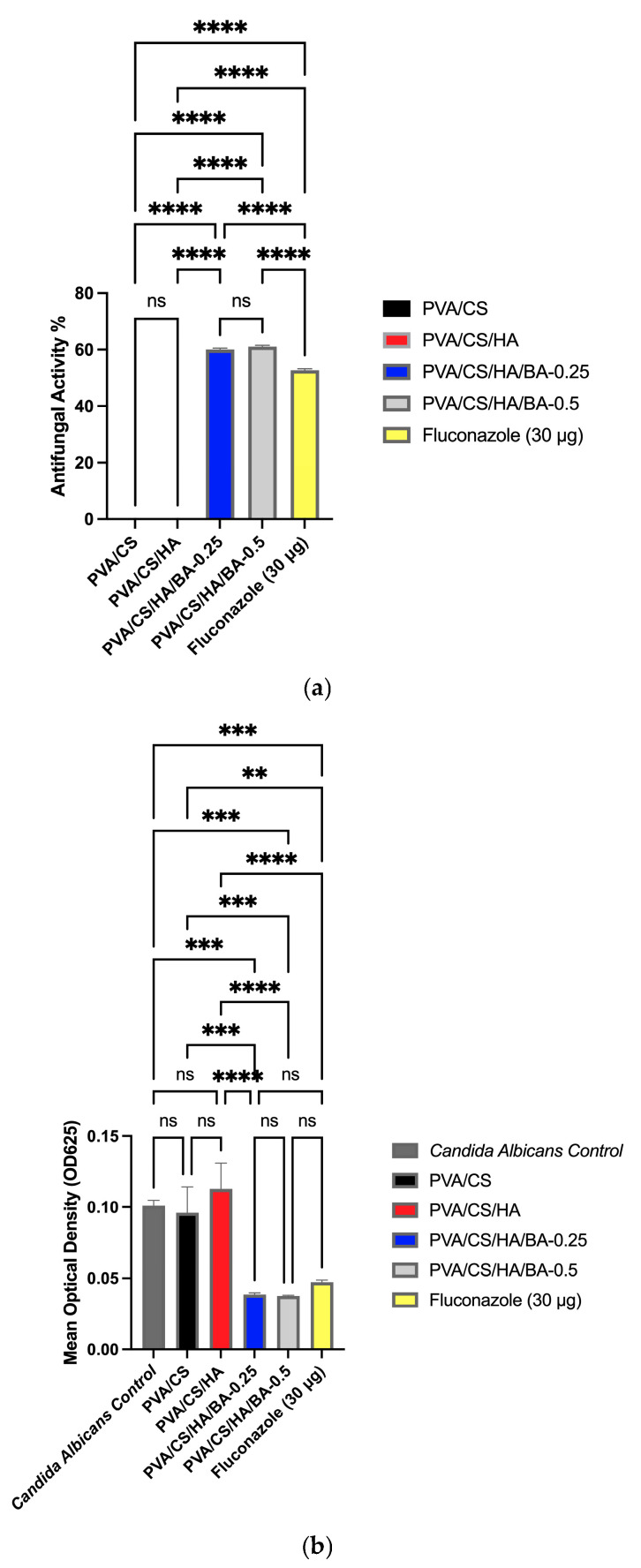
Antifungal activity of the electrospun PVA/CS, PVA/CS/HA, and BA-incorporated scaffolds against *Candida albicans* evaluated by the broth dilution method: (**a**) percentage of growth inhibition and (**b**) mean optical density (OD625). Fluconazole (30 µg) was utilized as an established positive control for comparison. All data are presented as mean ± SD (*n* = 3). Statistical analysis was performed using one-way ANOVA, followed by Tukey’s post hoc test. Asterisks indicate significant differences: **** *p* < 0.0001 compared to the untreated *Candida* control; *** *p* < 0.001, ** *p* < 0.01 indicate levels of significance. ‘ns’ (non-significant) denotes *p* > 0.05, indicating comparable efficacy between BA-containing scaffolds and the Fluconazole group.

In this study, the antifungal activity of nanofibers developed for bone tissue engineering applications on *Candida albicans* was evaluated via the broth dilution method. To provide a benchmark for comparison, both a negative control (*Candida* control) and a positive control (Fluconazole, 30 µg) were utilized. It was observed that while the untreated *Candida* control and pure PVA/CS/HA scaffolds showed maximum fungal growth (*OD*_625_), the addition of BA resulted in a dramatic and significant inhibition of *C. albicans* growth (*p* < 0.0001) (Figure 8). Specifically, the statistical analysis confirmed that BA-containing groups reached growth inhibition rates of approximately 60% for 0.25 wt.% BA and 61% for 0.5 wt.% BA groups.

Notably, the inhibition rates achieved by both 0.25 and 0.5 wt.% BA-containing nanofibers were found to be statistically comparable to the commercial antifungal agent Fluconazole (*p* > 0.05). This suggests that the developed scaffolds offer potent prophylactic protection that is similar to established standards. In the literature, BA is known to have this potent antifungal effect by disrupting fungal cell membrane integrity [81,82,83,84]. The use of materials with these biological effects can accelerate the treatment process and increase healing rates. Nanofibers are an important development area in nanotechnology for bone tissue engineering due to their cytocompatibility and biodegradability. Consequently, the antifungal effects of PVA/CS/HA/BA-0.25 nanofibers offer a promising alternative for bone tissue engineering that can be used to control infection in bone tissue and accelerate healing processes without the need for systemic drug administration.

The electrospinning process utilized in this study demonstrates high potential for industrial scalability. Furthermore, the GA vapor cross-linking provides the necessary structural stability to withstand standard sterilization processes, which is a critical requirement for future translational research and bone tissue engineering applications.

## 4. Conclusions

In the present study, multifunctional electrospun nanofiber scaffolds consisting of PVA/CS/HA/BA nanofibers have been successfully prepared with an optimized solution processing method to prevent pre-cross-linking reactions between PVA and BA. Detailed analysis not only confirmed that the simultaneous incorporation of HA and lower amounts of BA was advantageous in optimizing the nanofiber system’s effectiveness but also that HA and BA are well dispersed into the matrices of the scaffolds. Apart from these results of the SEM-EDS analysis, FT-IR spectra further demonstrated that both target functional groups, and borate-ester conversions associated with BA are preserved. DSC analysis demonstrated that HA enhanced thermal stability but introduced transitions in glass with multi-stage decomposition due to BA.

From the mechanical analysis, it was found that added HA greatly improved the tensile strength and flexibility. Although both concentrations of BA introduced a slight decrease in elongation properties, the biomechanical integrity was only marginally affected in the case of 0.25 wt.% BA. The cytocompatibility evaluation using hFOB cells confirmed that HA integration provided a supportive microenvironment for osteoblast growth, resulting in significantly increased relative proliferation over the 7-day culture period. Although both HA and BA influenced hFOB cells negatively, only 0.5 wt.% BA was toxic to hFOB cells. In fact, biocides should not pose toxic effects. In other words, biocides should not impair or obstruct biological function. Accordingly, based upon its biocidal property against *C. albicans*, biocidal agents similar to 0.25 wt.% BA should be considered.

Together, these outcomes indicate that PVA/CS/HA/0.25BA has the best values for both structural stability and heat treatment resistance. From its biological characteristics, cytotoxicity testing and antifungal analysis indicate good compatibility. These are crucial, because tissue-engineered scaffolds are expected to not only function biologically but also structurally. Based on these properties and characteristics, PVA/CS/HA/0.25BA can thus emerge as a promising candidate for translation to tissue engineering applications targeting potentially infected bones.

## Figures and Tables

**Table 1:** Compositions of prepared solutions of PVA/CS/HA/BA systems.

Solution	PVA (*w*/*v*%)	HA (*w*/*v*)	CS (*w*/*v*)	BA (*w*/*vt*)	TX-100 (*w*/*vt*)
PVA-CS	10	–	1	–	–
PVA-CS-HA	10	0.5	1	–	–
PVA-CS-HA-BA (0.25 BA)	10	0.5	1	0.25	1
PVA-CS-HA-BA (0.5 BA)	10	0.5	1	0.5	1

**Table 2:** Process parameters for electrospinning of PVA/CS/HA/BA nanofibers.

Solution	Voltage (kV)	Flow Rate (mL·h^−1^)	Distance (cm)	Spinning Duration (h)	Observation/Justification
PVA/CS	25	1.0	12	3	Stable Taylor cone; Baseline
PVA/CS/HA	25	1.0	12	3	Stable jet; Homogeneous HA
PVA/CS/HA/0.25BA	27	1.6	12	3	Increased for viscosity/viscoelasticity adaptation
PVA/CS/HA/0.5BA	27	1.6	12	3	Increased to prevent needle clogging and stabilize jet

**Table 3:** FTIR vibrational assignments and intensity analysis.

Wavenumber (cm^−1^)	Assignment	Component	Intensity Observation
3300	O–H/N–H stretching	PVA/CS	Broadens with BA addition [60,61]
2869–2989	C–H stretching	PVA/CS	Stable polymer backbone [62,63]
1592–1650	Amide I and II	CS	Confirms CS presence [64,65]
1365–1367	B–O (Borate-ester)	BA	Increases from 0.25 to 0.5% BA [48,66]
1030–1134	PO_4_^3−^ stretching	HA	Validates HA integration [11,12,56]
560–600	PO_4_^3−^ bonding	HA	Confirms HA crystallinity [11,65,67]

## Data Availability

The original contributions presented in this study are included in the article. Further inquiries can be directed to the corresponding author.

## References

[1] Santin M. (2009). 14—Bone tissue engineering. Bone Repair Biomaterials.

[2] Ma P., Wu W., Wei Y., Ren L., Lin S., Wu J. (2021). Biomimetic gelatin/chitosan/polyvinyl alcohol/nano-hydroxyapatite scaffolds for bone tissue engineering. Mater. Des..

[3] Salim S.A., Loutfy S.A., El-Fakharany E.M., Taha T.H., Hussien Y., Kamoun E.A. (2021). Influence of chitosan and hydroxyapatite incorporation on properties of electrospun PVA/HA nanofibrous mats for bone tissue regeneration: Nanofibers optimization and in-vitro assessment. J. Drug Deliv. Sci. Technol..

[4] Wongsawichai K., Kingkaew A., Pariyaisut A., Khondee S., Kingkaew E., Pariyaisut E. (2019). Porous hydroxyapatite/chitosan/carboxymethyl cellulose scaffolds with tunable microstructures for bone tissue engineering. Key Eng. Mater..

[5] Gavilanes-Martínez M.A., Coral-Garzón A., Cáceres D.H., García A.M. (2021). Antifungal activity of boric acid, triclosan and zinc oxide against different clinically relevant *Candida* species. Mycoses.

[6] Liu Q., Liu Z., Zhang C., Xu Y., Li X., Gao H. (2021). Effects of 3% Boric Acid Solution on Cutaneous *Candida albicans* Infection and Microecological Flora Mice. Front. Microbiol..

[7] De Seta F., Schmidt M., Vu B., Essmann M., Larsen B. (2008). Antifungal mechanisms supporting boric acid therapy of *Candida* vaginitis. J. Antimicrob. Chemother..

[8] Ahtzaz S., Sher Waris T., Shahzadi L., Anwar Chaudhry A., Ur Rehman I., Yar M. (2019). Boron for tissue regeneration-it’s loading into chitosan/collagen hydrogels and testing on chorioallantoic membrane to study the effect on angiogenesis. Int. J. Polym. Mater. Polym. Biomater..

[9] Ghiska R., Debie M.P., Albab M.F., Alfata R., Sofyan N., Yuwono H. (2018). The effect of HA addition on the mechanical properties of PVA/CS biomaterials for bone scaffold application. AIP Conference Proceedings.

[10] Abazari M.F., Nejati F., Nasiri N., Khazeni Z.A.S., Nazari B., Enderami S.E., Mohajerani H. (2019). Platelet-rich plasma incorporated electrospun PVA–CS–HA nanofibers accelerate osteogenic differentiation and bone reconstruction. Gene.

[11] Satpathy A., Pal A., Sengupta S., Das A., Hasan M., Ratha I., Barui A., Bodhak S. (2019). Bioactive nano-HA doped electrospun PVA–CS composite nanofibers for bone tissue engineering applications. J. Indian Inst. Sci..

[12] Januariyasa I.K., Ana I.D., Yusuf Y. (2020). Nanofibrous poly(vinyl alcohol)/CS containing carbonated HA nanoparticles scaffold for bone tissue engineering. Mater. Sci. Eng. C.

[13] Seeman E., Delmas P.D. (2006). Bone quality: The material and structural basis of bone strength and fragility. N. Engl. J. Med..

[14] Feng W., Li D., Zang J., Fu L. (2017). Biomechanical comparison of xenogeneic bone material treated with different methods. Xenotransplantation.

[15] Turner C.H. (2002). Biomechanics of bone: Determinants of skeletal fragility and bone quality. Osteoporos. Int..

[16] Sorushanova A., Delgado L.M., Wu Z., Shologu N., Kshirsagar A., Raghunath R., Mullen A.M., Bayon Y., Pandit A., Raghunath M. (2019). The collagen superfamily: From biosynthesis to advanced biomaterial development. Adv. Mater..

[17] Brun V., Guillaume C., Mechiche-Alami S., Josse J. (2014). CS/HA hybrid scaffold for bone tissue engineering. Bio-Medical. Mater. Eng..

[18] Gaweł J., Milan J., Żebrowski J., Płoch D., Stefaniuk I., Kus-Liśkiewicz M. (2023). Biomaterial composed of CS, riboflavin, and HA for bone tissue regeneration. Sci. Rep..

[19] Pineda-Castillo S., Bernal-Ballén A., Bernal-López C., Segura-Puello H., Nieto-Mosquera D., Villamil-Ballesteros A., Muñoz-Forero D., Munster L. (2018). Synthesis and characterization of poly(vinyl alcohol)–CS–HA scaffolds: A promising alternative for bone tissue regeneration. Molecules.

[20] Firnanelty S., Sugiarti S. (2017). Charlena Synthesis of HA–CS–PVAcomposite as injectable bone substitute material Rasayan. J. Chem..

[21] Mu X., Zheng W., Xiao L., Zhang W., Jiang X. (2013). Engineering a 3D vascular network in hydrogel for mimicking a nephron. Lab Chip.

[22] Gezer A.H., Acar A. (2020). Effects of boron compounds and ozonated olive oil on experimental Microsporum canis infection in rats. Turk. J. Veter. Anim. Sci..

[23] Kapukaya R., Ciloglu O. (2020). Treatment of chronic wounds with polyurethane sponges impregnated with BA particles: A randomised controlled trial. Int. Wound J..

[24] Tort S., Acartürk F. (2020). Investigation of propolis and boron containing disinfectants and comparison with WHO-recommended formulation against COVID-19. Gazi Med. J..

[25] Sayin Z., Ucan U.S., Sakmanoglu A. (2016). Antibacterial and antibiofilm effects of boron on different bacteria. Biol. Trace Elem. Res..

[26] Price C.J., Marr M.C., Myers C., Seely J.C., Heindel J.J., Schwetz B.A. (1996). The developmental toxicity of BA in rabbits. Toxicol. Sci..

[27] Erickson A.E., Edmondson D., Chang F.C., Wood D., Gong A., Levengood S.L., Zhang M. (2015). High-throughput and high-yield fabrication of uniaxially-aligned chitosan-based nanofibers by centrifugal electrospinning. Carbohydr. Polym..

[28] Sulutaş R.B., Cesur S., Seyhan S.A., Alkaya D.B., Şahin A., Ekren N., Gündüz O. (2024). Electrospun amygdalin and *Inula helenium* extract-loaded PLA/PVP nanofibrous patches for colon cancer treatment: Fabrication, characterization and antitumour effect. J. Drug Deliv. Sci. Technol..

[29] Das R., Burbery N.J. (2013). Trends and developments in the manufacturing of polymer nanofibrils with the electrospinning technique. Appl. Mech. Mater..

[30] Li Y., Bou-Akl T. (2016). Electrospinning in tissue engineering. Electrospinning—Material, Techniques, and Biomedical Applications.

[31] Paaver U., Heinämäki J., Laidmäe I., Lust A., Kozlova J., Sillaste E., Kirsimäe K., Veski P., Kogermann K. (2015). Electrospun nanofibers as a potential controlled-release solid dispersion system for poorly water-soluble drugs. Int. J. Pharm..

[32] Xue J., Wu T., Dai Y., Xia Y. (2019). Electrospinning and electrospun nanofibers: Methods, materials, and applications. Chem. Rev..

[33] Nokhasteh S., Molavi M.A., Ghayeni M.K., Avalshahr A.S. (2019). Preparation of PVA/CS samples by electrospinning and film casting methods and evaluating the effect of surface morphology on their antibacterial behavior. Mater. Res. Express.

[34] Koosha M., Mirzadeh H. (2015). Electrospinning, mechanical properties, and cell behavior study of CS/PVA nanofibers. J. Biomed. Mater. Res. Part A.

[35] Sundhamurti D., Vasanthan S.K., Kuppan P., Krishnan M.U., Sethuraman S. (2012). Electrospun nanostructured CS–poly(vinyl alcohol) scaffolds: A biomimetic extracellular matrix as dermal substitute. Biomed. Mater..

[36] Gadhave R.V., Mahanwar P.A., Gadekar P.T. (2019). Study of cross-linking between BA and different types of PVA adhesive. Open J. Polym. Chem..

[37] Araújo E.S., Nascimento M.L.F., de Oliveira H.P. (2013). Influence of Triton X-100 on PVA fibres production by the electrospinning technique. Fibres Text. East. Eur..

[38] Işık A.F., San Keskin N.O., Ulçay Y. (2018). Synthesis and in vitro antimicrobial characterization of boron–PVA electrospun nanofibers. J. Text. Inst..

[39] Matabola K.P., de Vries A.R., Luyt A.S., Kumar R. (2011). Studies on single polymer composites of poly(methyl methacrylate) reinforced with electrospun nanofibers with a focus on their dynamic mechanical properties. Express Polym. Lett..

[40] Zhang L., Tsuzuki T., Wang X. (2010). Preparation and characterization of cellulose nanofiber film. Mater. Sci. Forum.

[41] Kimura N., Kim H., Kim B., Lee K., Kim I.S. (2010). Molecular orientation and crystalline structure of aligned electrospun nylon-6 nanofibers: Effect of gap size. Macromol. Mater. Eng..

[42] Philip P., Jose E.T. (2025). Studies of interaction of methylene blue with pure and structurally modified electrospun poly(methyl methacrylate) nanofibers. Environ. Prog. Sustain. Energy.

[43] Kang H.K., Oh H.J., Kim J.Y., Kim H.Y., Choi Y.O. (2021). Effect of process control parameters on the filtration performance of PAN–CTAB nanofiber/nanonet web combined with meltblown nonwoven. Polymers.

[44] Cho Y.S., Yoon H., Jin S.G. (2024). Novel *Saccharomyces cerevisiae*-loaded polyvinylpyrrolidone/SiO_2_ nanofiber for wound dressing prepared using electrospinning method. Materials.

[45] Kai D., Prabhakaran M.P., Stahl B., Eblenkamp M., Wintermantel E., Ramakrishna S. (2012). Mechanical properties and in vitro behavior of nanofiber–hydrogel composites for tissue engineering applications. Nanotechnology.

[46] Venugopal J.R., Low S., Choon A.T., Ramakrishna S. (2007). Interaction of cells and nanofiber scaffolds in tissue engineering. J. Biomed. Mater. Res. Part B Appl. Biomater..

[47] Mao X., Bai Y., Yu J., Ding B. (2016). Flexible and highly temperature-resistant polynanocrystalline zirconia nanofibrous membranes designed for air filtration. J. Am. Ceram. Soc..

[48] Uslu I., Daştan H., Altaş A., Yayli A., Atakol O., Aksu M.L. (2017). Preparation and characterization of PVA/boron polymer produced by an electrospinning technique. e-Polymers.

[49] Özcan M., Kaya C., Kaya F. (2023). An optimization study for electrospun borate ester nanofibers as lightweight, flexible, and affordable neutron shields for personal protection. Macromol. Mater. Eng..

[50] Vu T.H.N., Morozkina S.N., Uspenskaya M.V. (2022). Study of the nanofibers fabrication conditions from the mixture of poly(vinyl alcohol) and CS by electrospinning method. Polymers.

[51] Hong X., He J., Zou L., Wang Y., Li Y.V. (2021). Preparation and characterization of high-strength and high-modulus PVA fiber via dry-wet spinning with cross-linking of BA. J. Appl. Polym. Sci..

[52] Ilhan E., Cesur S., Sulutaş R.B., Pilavci E., Dalbayrak B., Kaya E., Arisan E.D., Tinaz G.B., Sengor M., Kijeńska-Gawrońska E. (2022). The role of multilayer electrospun poly(vinyl alcohol)/gelatin nanofibers loaded with fluconazole and cinnamaldehyde in the potential treatment of fungal keratitis. Eur. Polym. J..

[53] Mansur H.S., Sadahira C.M., Souza A.N., Mansur A.A.P. (2008). FTIR spectroscopy characterization of poly(vinyl alcohol) hydrogel with different hydrolysis degrees and chemically crosslinked with glutaraldehyde. Mater. Sci. Eng. C.

[54] Varma R., Vasudevan S. (2020). Extraction, characterization and antimicrobial activity of CS from horse mussel *Modiolus modiolus*. ACS Omega.

[55] Rajasekaran S., Kungumaraj K.K., Mani D., Mani P. (2024). CS/guar gum–graphene oxide porous scaffolds for tissue engineering applications. Mater. Today Commun..

[56] Sistani S., Asgharzade S., Arab S., Bahraminasab M., Soltani-Fard E. (2025). Fabrication and evaluation of a host–guest polylactic acid/gelatin–HA–blueberry scaffold for bone regeneration. J. Orthop. Surg. Res..

[57] Lefèvre G. (2023). Determination of isotopic ratio of boron in BA solutions by ATR–FTIR spectroscopy. J. Radioanal. Nucl. Chem..

[58] Choe S., You S., Park K., Kim Y., Park J., Cho Y., Seo J., Yang H., Myung J. (2024). BA-crosslinked poly(vinyl alcohol): Biodegradable, biocompatible, robust, and high-barrier paper coating. Green Chem..

[59] Prosanov I.Y., Abdulrahman S.T., Thomas S., Bulina N.V., Gerasimov K.B. (2018). Complex of PVA with BA: Structure and use. Mater. Today Commun..

[60] da Mata G.C., Morais M.S., de Oliveira W.P., Aguiar M.L. (2022). Composition Effects on the Morphology of PVA/Chitosan Electrospun Nanofibers. Polymers.

[61] Sánchez-Téllez D.A., Baltierra-Uribe S.L., Vidales-Hurtado M.A., Valdivia-Flores A., García-Pérez B.E., Téllez-Jurado L. (2024). Novel PVA–Hyaluronan–Siloxane Hybrid Nanofiber Mats for Bone Tissue Engineering. Polymers.

[62] Jiamboonsri P., Sangkhun W., Wanwong S. (2024). Methyl Gallate and Amoxicillin-Loaded Electrospun Poly(vinyl alcohol)/Chitosan Mats: Impact of Acetic Acid on Their Anti-Staphylococcus aureus Activity. Polymers.

[63] Ceylan S., Dimmock R., Yang Y. (2023). Development of Boron-Containing PVA-Based Cryogels with Controllable Boron Releasing Rate and Altered Influence on Osteoblasts. Polymers.

[64] Çay A., Miraftab M., Kumbasar E.P.A. (2014). Characterization and swelling performance of physically stabilized electrospun poly(vinyl alcohol)/chitosan nanofibers. Eur. Polym. J..

[65] Iqbal N., Ganguly P., Yildizbakan L., Raif E.M., Jones E., Giannoudis P.V., Jha A. (2024). Chitosan Scaffolds from Crustacean and Fungal Sources: A Comparative Study for Bone-Tissue-Engineering Applications. Bioengineering.

[66] Takeno H., Shikano R., Kikuchi R. (2022). Mechanical performance of corn starch/poly(vinyl alcohol) composite hydrogels reinforced by inorganic nanoparticles and cellulose nanofibers. Gels.

[67] Srisang N., Maikaew J., Tambunlertchai S., Eiangmee O., Kamdenlek P., Manaspon C., Salem A.K., Phetpan K., Siriwan S., Sokjabok S. Optimizing Bone Scaffold Design Using Response Surface and Artificial Neural Network-Genetic Algorithm Methods with Biocompatibility Evaluation. https://ssrn.com/abstract=5340703.

[68] Cesur S., Ilhan E., Tut T.A., Kaya E., Dalbayrak B., Bosgelmez-Tinaz G., Arısan E.D., Gunduz O., Kijeńska-Gawrońska E. (2023). Design of cinnamaldehyde- and gentamicin-loaded double-layer corneal nanofiber patches with antibiofilm and antimicrobial effects. ACS Omega.

[69] Lemma S.M., Scampicchio M., Mahon P.J., Sbarski I., Wang J., Kingshott P. (2015). Controlled release of retinyl acetate from β-cyclodextrin-functionalized poly(vinyl alcohol) electrospun nanofibers. J. Agric. Food Chem..

[70] Peppas N.A., Merrill E.W. (1977). Crosslinked poly(vinyl alcohol) hydrogels as swollen elastic networks. J. Appl. Polym. Sci..

[71] Choo K., Ching Y.C., Chuah C.H., Julai S., Liou N.S. (2016). Preparation and characterization of PVA–CS composite films reinforced with cellulose nanofiber. Materials.

[72] Kanimozhi K., Khaleel Basha S., Sugantha Kumari V. (2016). Processing and characterization of chitosan/PVA and methylcellulose porous scaffolds for tissue engineering. Mater. Sci. Eng. C.

[73] Rinaudo M. (2006). Chitin and CS: Properties and applications. Prog. Polym. Sci..

[74] Pistone A., Iannazzo D., Celesti C., Piperopoulos E., Ashok D., Cembran A., Tricoli A., Nisbet D. (2019). Engineering of CS–HA–magnetite hierarchical scaffolds for guided bone growth. Materials.

[75] Radhakrishnan J., Gandham G.S.P.D., Sethuraman S., Subramanian A. (2015). Phase-induced porous composite microspheres sintered scaffold with protein–mineral interface for bone tissue engineering. RSC Adv..

[76] Dorozhkin S.V. (2010). Bioceramics of calcium orthophosphates. Biomaterials.

[77] Wang N., Zhao L., Zhang C., Li L. (2015). Water states and thermal processability of BA-modified poly(vinyl alcohol). J. Appl. Polym. Sci..

[78] Oktay B., Ciftci F., Erarslan A., Ahlatcıoğlu Özerol E. (2025). Dual-layer natamycin and boric-acid reinforced PVA/CS by 3D printing and electrospinning: Characterization and in vitro evaluation. Polymers.

[79] Yang D., Jin Y., Ma G., Chen X., Lu F., Nie J. (2008). Fabrication and characterization of CS/PVA with HA biocomposite nanoscaffolds. J. Appl. Polym. Sci..

[80] Rezaei A., Katoueizadeh E., Zebarjad S.M. (2023). Investigating the influence of zinc oxide nanoparticles morphology on the properties of electrospun polyvinyl alcohol/chitosan (PVA/CS) nanofibers. J. Drug Deliv. Sci. Technol..

[81] CLSI (2008). Reference Method for Broth Dilution Antifungal Susceptibility Testing of Yeasts.

[82] Iavazzo C., Gkegkes I.D., Zarkada I.M., Falagas M.E. (2011). Boric acid for recurrent vulvovaginal candidiasis: The clinical evidence. J. Women’s Heal..

[83] Syvolos Y., Salama O.E., Gerstein A.C. (2024). Constraint on boric acid resistance and tolerance evolvability in *Candida albicans*. Can. J. Microbiol..

[84] Güner P., Aşkun T., Er A. (2025). Antimicrobial potential of boron-containing compounds: Antibacterial, antifungal, and antimycobacterial activities. J. Boron.

